# Twelve-Week Yoga vs. Aerobic Cycling Initiation in Sedentary Healthy Subjects: A Behavioral and Multiparametric Interventional PET/MR Study

**DOI:** 10.3389/fpsyt.2021.739356

**Published:** 2021-10-18

**Authors:** June van Aalst, Lise Jennen, Koen Demyttenaere, Stefan Sunaert, Michel Koole, Jenny Ceccarini, Koen Van Laere

**Affiliations:** ^1^Nuclear Medicine and Molecular Imaging, Department of Imaging and Pathology, KU Leuven, Leuven, Belgium; ^2^Research Group Psychiatry, Neurosciences, University Psychiatric Center KU Leuven, Leuven, Belgium; ^3^Adult Psychiatry, University Hospitals Leuven, Leuven, Belgium; ^4^Translational MRI, Department of Imaging and Pathology, KU Leuven, Leuven, Belgium; ^5^Radiology, University Hospitals Leuven, Leuven, Belgium; ^6^Division of Nuclear Medicine, University Hospitals Leuven, Leuven, Belgium

**Keywords:** yoga, longitudinal interventional study, PET/MR imaging, FDG, synaptic density, indoor cycling

## Abstract

Interventional yoga studies with an active control group remain scarce and are important to clarify the underlying neurobiology. We conducted an interventional study in healthy controls using simultaneous positron emission tomography/magnetic resonance (PET/MR) imaging and psychometric scales. Thirty healthy, female volunteers (28.4 ± 8.4 years) participated and were randomly assigned to a 12-week yoga or indoor cycling intervention. Before and after the intervention, [^18^F]FDG and [^11^C]UCB-J PET was performed on a simultaneous GE Signa PET/MR with volumetric imaging. Psychometric scales were evaluated on affect, mindfulness, stress, worrying, self-compassion, and interoceptive awareness. Yoga subjects scored higher on interoceptive awareness compared to baseline (*p* < 0.001). Cognitive (*P* = 0.009) and overall cognitive functioning (*P* = 0.01) improved after the yoga intervention compared to the cycling group. We did not observe significant differences in glucose metabolism, synaptic density, or gray matter (GM) volume. The indoor cycling group did not show changes in psychometric variables, but significant increases in relative glucose metabolism were observed in the parahippocampal/fusiform gyrus and cerebellum (*P* < 0.001). In conclusion, 12 weeks of yoga practice has significant effects on interoceptive awareness and perceived cognitive function in starters. Longer interventions and/or higher frequency of yoga practice may be needed to detect cerebral metabolic and/or morphologic effects on the macroscopic level.

## Introduction

Yoga combines meditation (*dhyana*), physical postures (*asana*), and focused breathing (*pranayama*). It has become increasingly popular in the Western world as an approach to improve health and well-being (1) and has received more and more interest from a research perspective. Behavioral studies have shown that yoga can be an effective multi-component health intervention to reduce stress, increase physical fitness, and improve general well-being and quality of life (2, 3). Psychological dimensions improved by yoga include self and body awareness, coping capacity, stress, mindfulness, and self-compassion (2, 3). Besides behavioral studies, a limited number of imaging studies have investigated the effects of yoga on objective biomarkers in the brain. Different advanced neuroimaging techniques such as positron emission tomography (PET) and magnetic resonance (MR) imaging allow to investigate the biochemical, functional, and structural effects of yoga in a non-invasive way (4). However, since most studies so far have focused specifically on the meditational dimension of yoga, evidence for the combined tripartite effects is scarce. Based on imaging and physiological data, a leading hypothesis of an underlying neurobiological mechanism of yoga is that breathing exercises, meditation, and baroreflex-promoting poses induce a shift in the parasympathetic nervous system through activation of gamma aminobutyric acid (GABA) release through the vagal nerve (5, 6).

In cross-sectional studies, structural effects on gray matter (GM) volume have been described by MR imaging, with increased GM volume in the insular cortex and hippocampus as most consistently reported findings in (experienced) yoga practitioners (7, 8). It has been postulated that changes in GM volume might be the result of neuroplasticity (9). Few functional or molecular PET studies have been conducted in yoga practitioners. [^18^F]Fluorodeoxyglucose (FDG) PET imaging enables measurement of regional neuronal activity. In a recent [^18^F]FDG PET study in experienced yoga practitioners, we found a significant decrease in the limbic system compared to physically active but yoga-naive subjects (10). Such downregulation in metabolic activity could be due to GABA-mediated inhibition, development of more efficient brain metabolism, or a pre-existing phenotype in yoga practitioners. Therefore, a longitudinal study is warranted to clarify possible underlying mechanisms. Glucose metabolism is majorly determined by glutamate neurotransmission and neuron-astrocyte interactions (11). Furthermore, recently imaging of synaptic density has become available by means of PET radiotracers such as [^11^C]UCB-J. This ligand binds to the presynaptic vesicle protein 2A (SV2A) with high affinity and specificity, and is altered in several neuropsychiatric conditions (12–16). For the first time, this opens the possibility to investigate whether an intervention can induce neuroplasticity by axon sprouting and neurogenesis (9). Combined measurement of synaptic activity and synaptic density could therefore offer complimentary measures of brain function (17).

As the choice of an appropriate control group is critical to disentangle the impact of yoga practice on brain function without confounding factors (4, 18), we have chosen to use moderate-intensity indoor cycling as control intervention. Both interventions are practiced in group, can be guided by a skilled teacher, are of similar duration per exercise unit and can be metabolically matched. Also aerobic exercise induces beneficial psychological effects, such as increased self-esteem, self-satisfaction, confidence, and improved turmoil (19).

The aim of this study was to compare a 12-week yoga intervention vs. aerobic moderate intensity exercise with neuropsychological endpoints as well as longitudinal positron emission tomography/magnetic resonance (PET/MR) imaging of glucose metabolism, synaptic density and structural imaging. This was performed in yoga-naïve sedentary individuals to exclude effects of previous training.

## Materials and Methods

### Subjects

In total, 30 right-handed healthy sedentary female volunteers [*n* = 30; age: 28.4 ± 8.4 (SD) years] participated in the study. Subjects were in good health according to their medical history, physical examination, general laboratory test (blood and urine) screening, and general neuropsychological evaluation [Symptoms Checklist (SCL90), Beck's depression inventory (BDI) (20), and mini-mental state examination (MMSE)]. The main exclusion criteria consisted of a history of major internal disease, previous severe head trauma, a psychiatric disorder, and use of centrally acting drugs. All subjects were required to have a sedentary lifestyle, defined as doing <1 h of exercise a week the year prior to study participation. The study was approved by the local University Ethics committee (study number S59792—Belgian Registration Number B32220173162) and was conducted in full accordance with the latest version of the Declaration of Helsinki. All participants provided written informed consent before inclusion in the study.

### Study Design

The study design is reported in Supplementary Figure 1. All subjects underwent up to two PET/MR scans ([^18^F]FDG in all and [^11^C]UCB-J for most participants) at baseline and after 12 weeks of intervention. After the baseline scan, subjects were randomly assigned to either the yoga intervention group (*n* = 15) or an indoor cycling intervention group (*n* = 15) (physical blinded number picking by the subjects). The yoga group was planned to attend yoga classes for 12 weeks, twice a week with 60-min sessions. In addition, their regular exercise regimen (<1 h/per week) was allowed. All yoga sessions took place in the same studio in Leuven (Flowing Yoga, Mrs. K. Marent). Different yoga styles for beginners were allowed to the participant's choice, including easy flow, *prana vinyasa* easy flow, *ashtanga* basics, and *Yin* and *Yang* yoga. These yoga styles all included approximately the same time ratio of physical postures (±70%), breathing exercises (±25%, in between the different postures) and guided meditation (±5%).

Participants assigned to the cycling group had to attend 60 min of indoor cycling classes for 12 weeks, also twice a week (with regular exercise routine (<1 h of exercise/per week) allowed). At their first training, cycling group subjects had to perform an individual power level test, to determine their individual threshold power defined as 90% of their peak power. The cycling subjects then received the instruction to keep the mean power below 80% of their individually determined threshold during the cycling classes, in order to stay within aerobic conditions and to match the physical intensity level with the yoga intervention.

Both yoga and cycling sessions were registered by the yoga studio and sport center, respectively. Moreover, participants had to keep a diary to track their lessons. For both groups a minimum of 20 lessons was required to complete the study.

### Psychometric Evaluation

All participants completed a battery of psychometric questionnaires at baseline and post intervention. In line with the previously observed psychological effects of yoga (3), the following dimensions were sampled: affect (21), mindfulness (22–24), stress (2, 22), worrying (22), self-compassion (23), and interoceptive awareness (25). The specific scales sampled in this study included:

– *Multi-assessment interoceptive awareness* (MAIA) scale. The MAIA questionnaire measures interoceptive awareness, defined as the awareness of signals from the inside of the body and higher-order top down processes. In total eight subdimensions are measured: noticing, not-distracting, not-worrying, attention regulation, emotional awareness, self-regulation, body listening, and trust (26).
– *Leuven Affect and Pleasure Scale* (LAPS) (27). This scale offers a comprehensive assessment of negative and positive affect, hedonic tone, and independent variables on cognitive and overall functioning, evaluation of a meaningful live, and happiness.
– *Five-Facet Mindfulness Questionnaire* (FFMQ) (28). Mindfulness is defined as “paying attention in a particular way: on purpose, in the present moment, and non-judgmentally.” This questionnaire includes five factors that represent elements of mindfulness, including observing, describing, acting with awareness, non-judging of inner experience, and non-reactivity to inner experience.
– *Perceived Stress Scale* (PSS) (29). This psychological instrument is used to measure perception of stress.
– *Penn State Worry Questionnaire* (PSWQ) (30) to measure the trait of worry.
– *Self-Compassion Scale* (SCS) (31). Self-compassion is described as “being open to and moved by one's own suffering, experiencing feelings of caring and kindness toward oneself, taking an understanding, non-judgmental attitude toward one's inadequacies and failures, and recognizing that one's own experience is part of the common human experience.” This scale is a psychometrical measure of self-compassion and includes six subscales: self-kindness, self-judgment, common humanity, isolation, mindfulness, and over-identification.

### Image Acquisition

All PET and MR data were acquired on a simultaneous Signa time-of-flight (TOF) PET/MR scanner with fast Silicon photomultiplier detectors inside a 3T MR magnet (GE Healthcare, Chicago, IL, USA). Subjects fasted at least 3 h prior to [^18^F]FDG injection. Subjects received an intravenous bolus injection of [^18^F]FDG (at baseline: 118 ± 12 MBq and post-intervention: 119 ± 9 MBq) in supine position with a 20-min accumulation period in a quiet and dimly lit environment. During the accumulation period, subjects were asked to close their eyes but remain awake. Subsequently, a static 30-min [^18^F]FDG PET/MR scan was acquired. [^18^F]FDG (Glucogast^TM^, UZ Leuven, Belgium) was produced in-house according to an approved manufacturing authorization, with a radiochemical purity >95%. After the [^18^F]FDG PET acquisition, a single venous blood sample was collected to measure blood glucose concentration and the remaining [^18^F]FDG radioactivity to calculate a simplified measure of absolute glucose consumption (Hunter method) as validated previously against absolute arterial spin labeling (10, 32).

Additionally, a subset of participants (*n* = 20; 10 in each group) received an additional SV2A PET scan using [^11^C]UCB-J. This subset was chosen randomly, and only based on the logistics (availability) of the [^11^C]UCB-J tracer production. The precursor was obtained from UCB and labeled on site under GMP standards with a radiochemical purity >95%, as described previously (33). Subjects received a bolus injection of 270 ± 60 MBq (specific activity 239 ± 133 GBq/μmol) and of 262 ± 61 MBq (specific activity 187 ± 73 GBq/μmol) at baseline and post-intervention, respectively. This bolus was administered at least 100 min prior to the [^18^F]FDG injection (with 20 min physical half-life the [^11^C]UCB-J activity was mostly decayed before [^18^F]FDG PET). Sixty minutes post-injection, a 30-min static [^11^C]UCB-J scan was acquired to be quantified using a reference tissue approach (33).

PET data were rebinned in six frames of 5 min, corrected for dead time, randoms, scatter, and time-offset (34). An MR-based attenuation correction (MRAC), based on zero-echo time (ZTE) MR images (3D radial acquisition; Flip Angle: 0.8°; Bandwidth: 62.5 kHz), was used for attenuation correction (35). Positron emission tomography images were reconstructed using OSEM (ordered subset expectation maximization; 28 subsets; 4 iterations) algorithm, including time of flight (TOF) information, resolution modeling, and an in-plane Gaussian post-smoothing with a FWHM (full width at half maximum) of 4.5 mm.

Simultaneous with the PET data acquisition, the following MR sequences were acquired [using an eight-channel high-resolution receiver head coil (GE Healthcare)]: 3D volumetric T1-weighted BRAVO (plane: sagittal; TE: 3.2 ms; TR: 8.5 ms; TI: 450 ms; flip angle: 12°; receiver bandwidth: 31.25 kHz; voxel size: 1 × 1 × 1 mm) and fluid-attenuated inversion recovery (FLAIR) 3D CUBE (TR: 8,500 ms, TE 130 ms, voxel size: 1 × 1 × 1.4 mm).

### Image Data Analysis

Both [^18^F]FDG and [^11^C]UCB-J PET data were analyzed on a voxelwise basis, using SPM12 (Statistical Parametric Mapping, Wellcome Department of Imaging Neuroscience, London, UK), and using a predefined volume-of-interest (VOI) approach (PMOD software v3.9, PMOD Inc., Zurich, Switzerland).

Reconstructed PET data were corrected for motion. Parametric standardized uptake value ratio (SUVR) images for [^11^C]UCB-J were generated, using the centrum semiovale (CS) as validated reference region in healthy volunteers (33, 36). For [^18^F]FDG PET regional cerebral metabolic rate of glucose (rCMRGlc) (mmol/l/min) maps, first blood glucose concentration was measured at the end of the scan and a venous blood sample was centrifuged for 5 min (4,000 rpm, 4°C) to measure the remaining tracer concentration in plasma (gamma counter; Perkin Elmer, 1480 WIZARD). A lumped constant of 0.65 was applied for all regions to calculate rCMRGlc values (32, 37). For two subjects (both in the yoga group at post-intervention), these maps could not be generated due to technical errors in plasma analysis and blood sample withdrawal, respectively. These two subjects were therefore excluded from the rCMRGLc data analysis.

All post-intervention [^18^F]FDG and [^11^C]UCB-J parametric PET maps were first co-registered to their respective baseline images. Subsequently, all PET images were co-registered to the subject's own T1-weighted MR image and spatially normalized to the Montreal Neurological Institute (MNI) space using a non-linear normalization with a DARTEL algorithm (SPM12). To reduce noise at the voxel level and account for gyral variations, PET images were additionally smoothed using a Gaussian FWHM of 8 mm. To exclude extracerebral activity, a relative threshold of 80% of the mean and an implicit CSF and GM mask was used.

Voxel-based findings were corroborated with a predefined VOI analysis using the N30R83 Hammers probabilistic atlas and AAL-merged in PMOD (38, 39), as the AAL-atlas allows for a more detailed delineation of the entire brainstem (VOIs for the medulla, pons, and midbrain). To reduce dimensionality and avoid type II errors, the standard 83 VOIs were merged into 12 larger, bilateral VOIs: FCx, frontal cortex; ACCx, anterior cingulate cortex; PCCx, posterior cingulate cortex; LTL, lateral temporal lobe; MTL, medial temporal lobe; PCx, parietal cortex; OCx, occipital cortex; Str, striatum; Thal, thalamus; ICx, insular cortex; Cbl, cerebellum; Bs, brainstem.

For the voxel-based morphometry (VBM) analysis, the Computational Anatomy Toolbox (CAT12) (40) implemented in SPM12 was used. All individual T1-weighted MR images were segmented into GM, white matter (WM), and cerebrospinal fluid (CSF), spatially normalized using the DARTEL algorithm and modulated with the Jacobian warp parameters. After pre-processing, GM images were smoothed with a Gaussian kernel of 8 mm. An absolute threshold masking of 0.1 to avoid edge effects around borders between GM, WM, and CSF was used.

### Statistical Analyses

Statistical analyses were conducted using Prism (v5, GraphPad, San Diego, USA) or SPSS (v26, IBM, Corporation, Chicago, Illinois). *P*-values were considered significant at an alpha level of 0.05. For the psychometric questionnaires, data were analyzed in a repeated ANOVA design (interaction effect group × time), followed by *post-hoc* between-group unpaired *t*-tests (yoga vs. indoor cycling, at baseline and post-intervention) and within-group paired *t*-tests (baseline vs. post-intervention, in the yoga and indoor cycling group). In SPM, both PET targets and the T1-weighted images (VBM) were explored in a flexible factorial design to investigate interaction effects and in a 2 × 2 design; *post-hoc* between-group unpaired *t*-tests (yoga vs. indoor cycling, at baseline and post-intervention) and within-group paired *t*-tests (baseline vs. post-intervention, in the yoga and indoor cycling group). Both absolute (parametric rCMRGlc images and SUVR [^11^C]UCB-J images) and relative ([^18^F]FDG uptake and [^11^C]UCB-J, normalized to global [^18^F]FDG uptake and global [^11^C]UCB-J binding in GM, respectively) were analyzed. SPM data were analyzed at a voxel-level *P*_height_ < 0.001, cluster extent threshold *k*_*E*_ = 237 voxels (corresponding to a size of 0.8 cm^3^; applied voxel size = 1.5 × 1.5 × 1.5 mm), and cluster-level *P*_FWE_ < 0.05. Total intracranial volume was used as covariate for the VBM SPM group analysis. Correlations between significant effects on brain regions and the psychometric scores were explored.

## Results

### Subject Characteristics

In total 33 subjects were initially included and scanned at baseline. Two subjects withdrew and one was excluded after the baseline scan due to significant WM lesions due to a delivery trauma at birth. After randomization (paper picking of numbers 1 or 2 by the subjects), a small but significant age difference [31.8 ± 9.8 years (yoga, range 22–51 years) vs. 24.9 ± 5.1 years (cycling, range 19–38 years), *P* = 0.02] was present between both groups. This difference was neglected as for FDG PET, SV2A PET density and structural MR imaging no significant age effect between 20 and 50 years is known (41–45). Also, although both female and male subjects were eligible in the study, only female subjects were included (Table 1). In the yoga group, the average number of lessons attended was 21.1 ± 1.2 (range 20–24); similar to the cycling group: 21.4 ± 1.4 (range 20–24), *P* = 0.49.

**Table 1:** Subject demographics and study-related variables.

	**Yoga group**	**Cycling group**	***P*-value**
	**(*n* = 15)**	**(*n* = 15)**	
Age	31.8 ± 9.8	24.9 ± 5.1	0.02
Sex (F/M)	15/0	15/0	
Activity level (hrs/wk)	0.5 ± 0.4	0.6 ± 0.5	0.98
Pre-PET scan sober glycaemia (mg/dl)	86.5 ± 6.1	85.5 ± 6.1	0.68
BMI (kg/m^2^)	23.4 ± 2.3	22.5 ± 2.4	0.30
Educational level			0.66
High school	2 (13.3%)	4 (26.7%)	
Bachelor degree	8 (53.3%)	7 (46.7%)	
Master degree	5 (13.3%)	4 (26.7%)	
BDI	3 (0–8)	1 (0–8)	0.32
MMSE	30 (29,30)	29 (29,30)	0.07
Nr of attended classes (out of max 24)	21.1 ± 1.2	21.4 ± 1.4	0.49

*BDI, beck depression inventory; BMI, body mass index; F, female; M, male; MMSE, mini-mental state examination. Data are presented as mean ± standard deviation for continuous variables, median (min-max) for integer variables and frequency (%)*.

### Psychometric Scales

**Figure 1:**
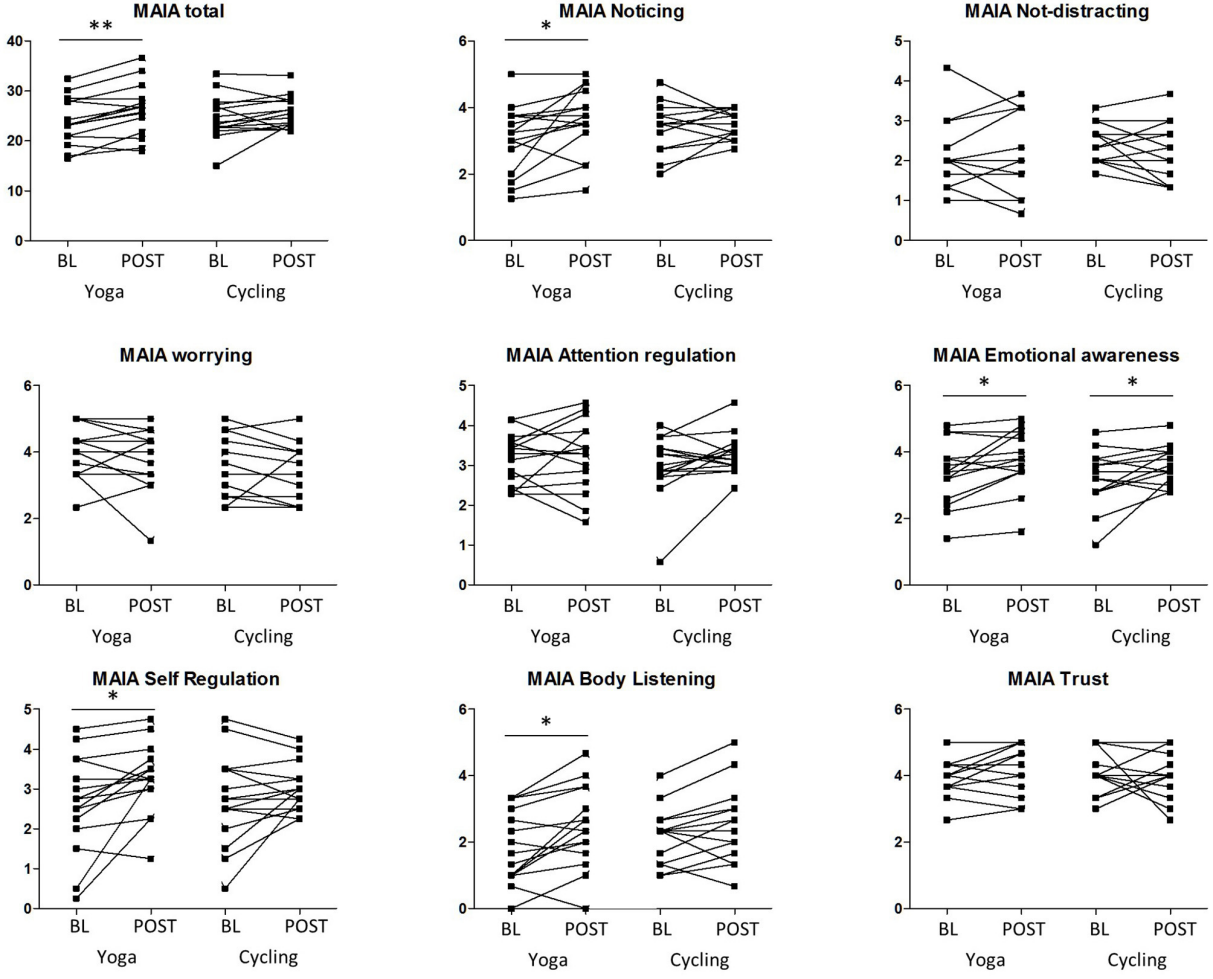
Results of the multidimensional assessment of interoceptive awareness (MAIA) scale and subscales of both groups (yoga and cycling), at baseline (BL) and post-intervention (POST). Significant results of the paired tests are indicated with * (*P* < 0.05, uncorrected) and ** (*P* < 0.005, uncorrected).

For the psychometric scales, no significant interaction effect (group × timing) was found. However, a significant increase after yoga intervention was observed in the MAIA interoceptive awareness total score compared to baseline (26.2 ± 5.2 vs. 23.9 ± 4.7, *P* = 0.001 (uncorrected), remaining significant after Bonferroni correction for the number of scales) (Figure 1; Table 2). For the MAIA subscores, this increase was also reflected in the subdimensions “noticing” (*P* = 0.02 (uncorrected), Bonferroni uncorrected), “emotional awareness” (*P* = 0.009, uncorrected), “self-regulation” (*P* = 0.015, uncorrected), and “body listening” (*P* = 0.007, uncorrected). For the cycling group, a significant (*P* = 0.04, uncorrected) increase in the emotional awareness score was observed, but no overall effect on the global MAIA score.

**Table 2:** Psychometric results of the between and within group analyses.

	**Yoga**		**Cycling**		**Repeated** ***ANOVA*** ***P*-value**	**Within groups (paired)** **(intervention effect)**		**Between groups (unpaired)** **(group effect)**	
**Scale**	**Baseline**	**Post**	**Baseline**	**Post**	**Group × Time**	**Yoga**	**Cycling**	**Baseline**	**Post**
**LAPS**									
Positive affect	7.8(1.1)	7.9(1.3)	7.2(1.4)	7.3(1.2)	0.94	0.78	0.87	0.23	0.22
Negative affect	1.2(1.0)	2.0(1.6)	1.6(0.9)	2.2(1.5)	0.73	0.07	0.15	0.23	0.69
Hedonic tone	8.8(1.0)	8.6(1.1)	8.5(0.9)	8.1(1.1)	0.64	0.35	0.06	0.34	0.22
Independent variables:									
Cognitive functioning	8.1(1.7)	8.6(1.1)	7.9(1.5)	7.3(1.4)	0.06	0.20	0.18	0.66	0.009^a^
Overall functioning	8.7(1.3)	8.8(0.9)	8.2(1.3)	7.7(1.3)	0.26	0.84	0.19	0.28	0.01^a^
Meaningful life	8.5(1.0)	8.1(1.2)	7.4(2.2)	7.9(1.2)	0.30	0.21	0.52	0.10	0.56
Happiness	8.5(1.1)	8.3(1.2)	7.5(1.6)	7.7(1.5)	0.33	0.30	0.63	0.04	0.23
**FFMQ**	137.5(15.2)	138.9(18.9)	139.4(13.3)	140.9(13.9)	0.97	0.51	0.55	0.71	0.74
Observe items	24.5(5.4)	26.8(5.8)	25.9(4.4)	27.5(5.0)	0.54	0.04^a^	0.07	0.42	0.74
Describe items	28.67(5.4)	28.5(5.3)	30.1(5.5)	30.0(6.3)	0.96	0.81	0.90	0.47	0.48
Awareness items	29.7(5.4)	28.0(5.8)	29.3(4.7)	29.1(5.4)	0.35	0.09	0.87	0.86	0.59
Non-judge items	30.8(4.7)	30.9(4.9)	32.5(3.4)	32.4(4.0)	0.91	0.93	0.95	0.27	0.36
Non-react items	23.9(4.2)	24.7(3.5)	21.5(4.9)	21.9(4.5)	0.71	0.41	0.59	0.17	0.07
**PSS**	12.1(6.3)	12.6(5.4)	12.1(5.8)	12.9(5.4)	0.86	0.80	0.49	0.98	0.87
**PSWQ**	31.0(13.2)	30.1(14.9)	33.2(14.8)	36.6(15.2)	0.50	0.83	0.48	0.67	0.25
**SCS**	28.1(7.5)	27.2(6.0)	29.9(4.4)	28.0(4.3)	0.51	0.30	0.06	0.41	0.69
**MAIA**	23.9(4.7)	26.2(5.2)	24.8(4.4)	25.8(3.2)	0.18	0.001^b^	0.21	0.61	0.78
Noticing	3.0(1.0)	3.6(1.0)	3.5(0.8)	3.6(0.4)	0.07	0.02^a^	0.58	0.20	0.81
Not-distracting	2.1(0.8)	2.1(0.9)	2.4(0.5)	2.2(0.7)	0.39	0.88	0.17	0.25	0.72
Not-worrying	4.0(0.8)	3.8(0.9)	3.7(0.9)	3.6(0.9)	0.78	0.29	0.41	0.40	0.55
Attention regulation	3.2(0.6)	3.2(0.9)	3.0(0.8)	3.3(0.5)	0.45	0.68	0.14	0.52	0.94
Emotional awareness	3.4(0.9)	3.8(0.9)	3.3(0.9)	3.6(0.6)	0.90	0.009^a^	0.04^a^	0.69	0.56
Self-regulation	2.6(1.2)	3.3(0.9)	2.7(1.1)	3.0(0.6)	0.28	0.015^a^	0.40	0.82	0.40
Body listening	1.8(1.1)	2.5(1.2)	2.2(0.8)	2.5(1.1)	0.20	0.007^a^	0.10	0.33	0.96
Trusting	3.8(0.6)	4.0(0.8)	4.0(0.7)	4.0(0.7)	0.52	0.13	0.93	0.33	0.81

*BL, baseline; FFMQ, five facet mindfulness questionnaire; LAPS, Leuven affect and pleasure scale; MAIA, multi-assessment interoceptive awareness; PSS, perceived stress scale; PSWQ, Penn state worrying questionnaire; SCS, self-compassion scale*.

a *(significant) uncorrected*,

b *remains significant after Bonferroni correction*.

Furthermore, in the yoga subjects, scores on the “observe items” of the mindfulness FFMQ questionnaire increased significantly (*P* = 0.04, uncorrected) compared to baseline (Table 2). No significant intervention differences were observed for the PSS, PSWQ, and SCS.

For the between group analysis at baseline, both groups did not score significantly different on the psychometric scales. A significant difference between groups was observed after the intervention however, showing higher scores on “cognitive functioning” (*P* = 0.009, uncorrected) and “overall functioning” (*P* = 0.01, uncorrected) subscales of the LAPS affect and pleasure scale in the yoga group compared to the cycling group.

### Glucose Metabolism and Intervention Effects

**Figure 2:**
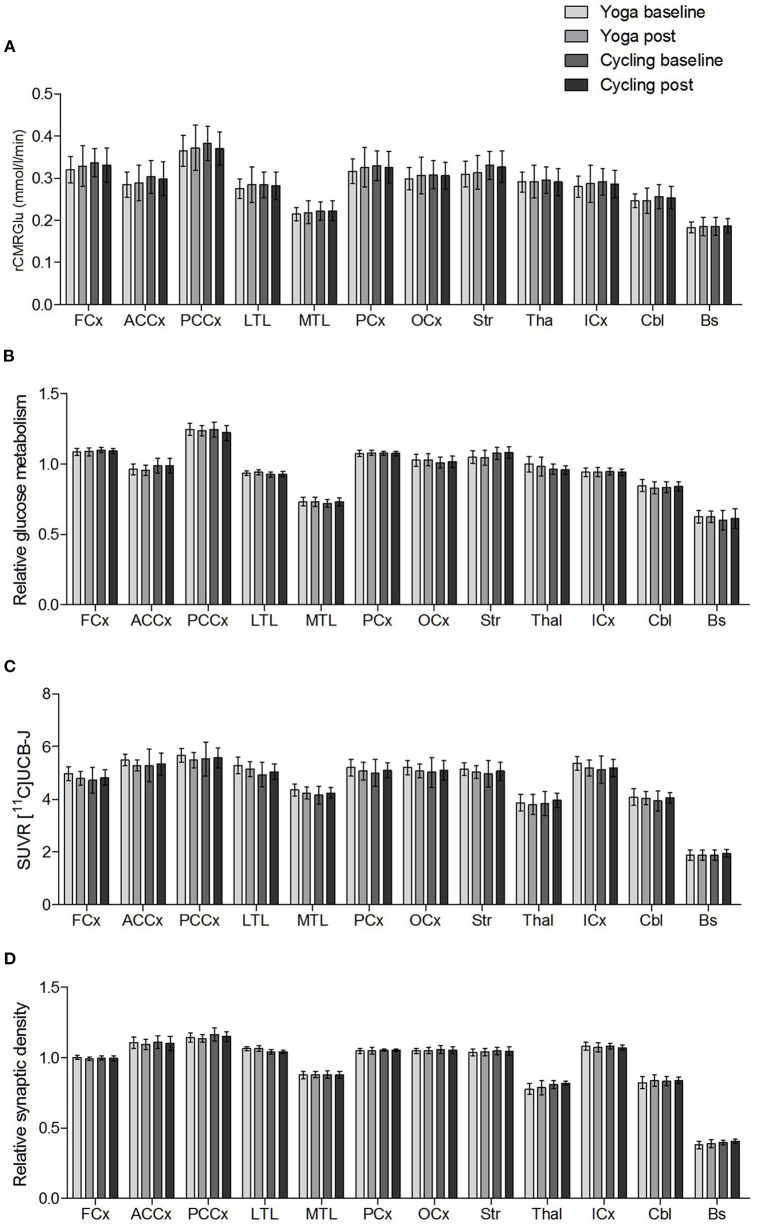
Regional mean **(A)** absolute glucose metabolism (rCMRGlu), **(B)** relative glucose metabolism, **(C)** SUVR [^11^C]UCB-J, and **(D)** relative synaptic density in the composite VOIs (FCx, frontal cortex; ACCx, anterior cingulate cortex; PCCx, posterior cingulate cortex; LTL, lateral temporal lobe; MTL, medial temporal lobe; PCx, parietal cortex; OCx, occipital cortex; Str, striatum; Thal, thalamus; ICx, insular cortex; Cbl, cerebellum; Bs, brainstem). Error bars represent one standard deviation.

**Figure 3:**
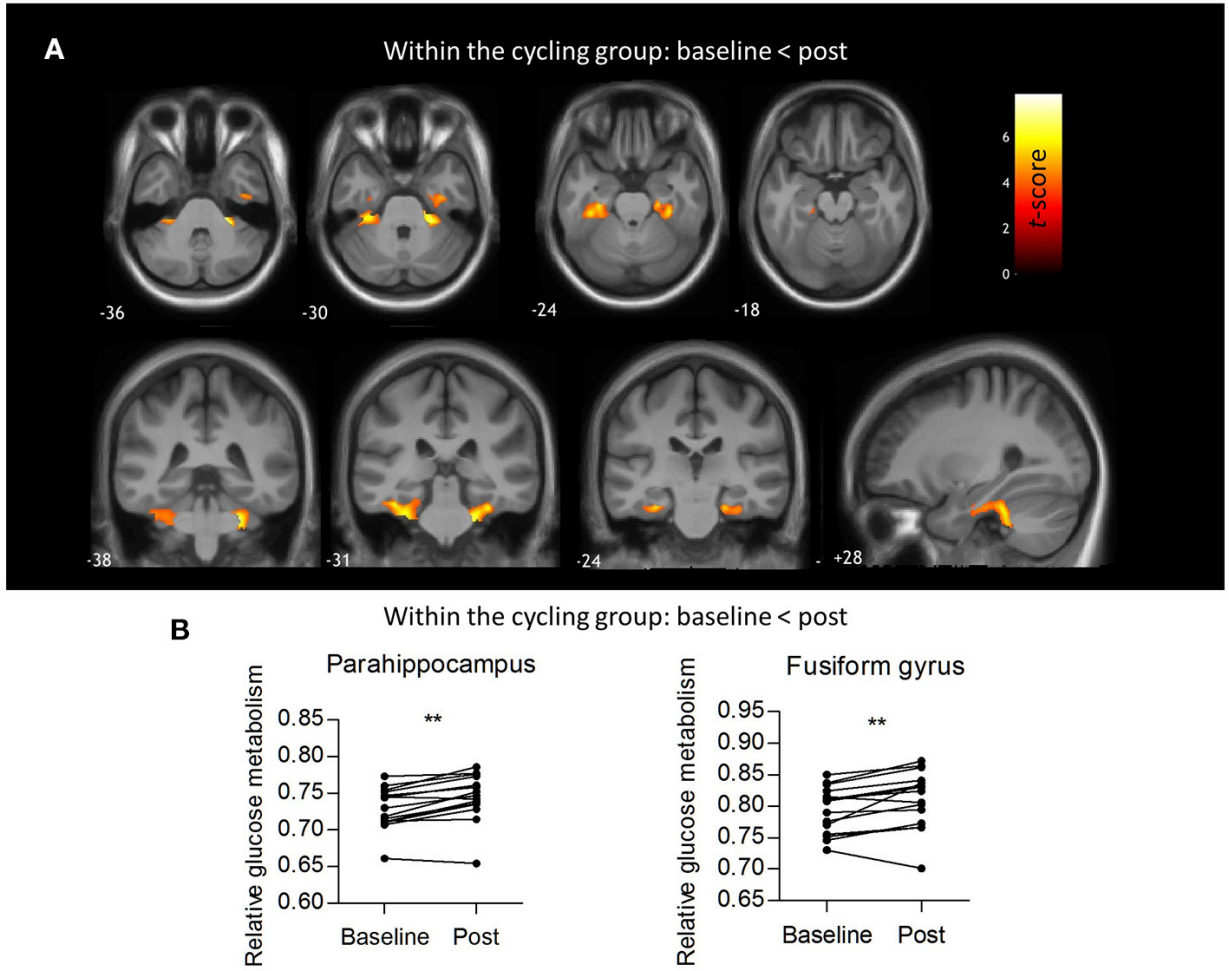
Within-groups analysis of relative glucose metabolism. **(A)** Voxel-based paired *t*-statistical map for the cycling group showing increased relative glucose metabolism post intervention compared to baseline. Evaluation at *P*_FWE_ < 0.05 corrected at cluster level, *P*_height_ < 0.001 uncorrected at voxel level, *K*_ext_ >0.8 cm^3^. The results are projected on the group's average 3D T1-weighted MR. **(B)** Regional relative glucose metabolism in the parahippocampus (+2.2%; *P* = 0.0002) and fusiform gyrus (+2.2%; *P* = 0.006), paired *t*-tests. **: P < 0.005.

No significant interaction effects were found. No significant differences in absolute glucose metabolism were found between both groups (at baseline and post-intervention), nor within groups (comparing the baseline vs. post-intervention condition). The mean absolute glucose metabolism values in the different composite VOIs are shown in Figure 2A. For relative glucose metabolism, normalized on total GM, no differences were found between groups Figure 2B. Within-group analyses showed no significant differences after the yoga intervention. However, for the indoor cycling group, the paired within-group showed significant increases in relative glucose metabolism in the cerebellum (region 4, 5, 6, and 10), fusiform gyrus, and parahippocampus with a peak effect in the right and left upper cerebellar gyrus (region 4/5), of +4.3 and +5.7%, respectively (Figure 3A; Supplementary Table 1). The VOI-based analysis confirmed this significant relative increased glucose metabolism in the parahippocampus (+2.2%; *P* = 0.0002) and fusiform gyrus (+2.2%; *P* = 0.006) (Figure 3B). Regional average relative glucose metabolism values in the different composite VOIs are given in Figure 2B. No significant correlations were found between the psychometric scores and increased regional relative glucose metabolism in the indoor cycling group.

### Synaptic Density and Intervention Effects

No significant interaction effects were found. The groups did not differ in synaptic density (*n* = 10 each), both in absolute (SUVR) as well as in relative terms. Neither intervention resulted in a demonstrable effect on regional synaptic density. The mean [^11^C]UCB-J SUVR and relative synaptic density VOI-based values for the baseline and post intervention conditions are shown in Figures 2C,D.

### Gray Matter Volume and Intervention Effects

No significant interaction effects were found. At baseline, no differences in GM volume values were found between both groups. Also, VBM did not show significant changes in GM volume after the 12-week yoga intervention nor after the cycling intervention.

## Discussion

Several cross-sectional imaging studies have shown that long-term yoga practice may lead to both structural and functional/metabolic alterations (7, 46). The objective of this (current) study was to determine in a longitudinal study whether behavioral and multimodal imaging biomarker change in sedentary healthy subjects starting either a yoga intervention vs. a physical matched intervention. Whereas, a clear effect on relevant behavioral changes was present, including interoceptive awareness and cognition scores, no significant imaging-based changes were found after this 12-week intervention.

For the psychometric scales, especially interoceptive awareness increased significantly after successful completion of a 12-week yoga intervention with more than 20 sessions. Previous studies have shown similar behavioral effects after mindfulness interventions, accompanied by increases in the MAIA subscales as well (26, 47). After a mindfulness intervention of 3 months, Bornemann et al. found significant overall increase in interoceptive awareness in healthy volunteers, with significant changes on self-regulation, attention regulation, emotional awareness, body listening, and trusting subscales. Also de Jong et al., investigating patients with chronic pain and comorbid depression, reported significant effects of an 8-week mindfulness intervention, on self-regulation, emotional awareness, and not-distracting subscales (26, 47). Effects on the self-regulation and emotional awareness subscales, also found in our study, thus show an overlapping effect of mindfulness and yoga-based interventions. Of interest, in our study specific “formal” guided meditation components represent only a minor part of the whole yoga lesson. During yoga practice, interoceptive awareness is addressed by drawing attention, feeling emotions, and bodily sensations to the present moment. This indicates that adding the dimensions of breathing techniques and meditation are needed to alter interoceptive awareness, as only exercise as shown in the control arm with indoor cycling, was unable to change this. In contrast, the FFMQ, a particular scale oriented toward mindfulness did not change significantly in our study. This is in contrast to previous interventional studies that had a stronger focus on additional mindfulness or yoga philosophy components such as Kripalu yoga emphasizing the cultivation of “witness consciousness” and compassion, and a combination of yin yoga and mindfulness (22, 24), compared to the yoga styles practiced in this study. Specific mindfulness-based interventions may be needed to improve self-compassion (48). Also, a significant relationship between class frequency or practice experience with mindfulness scores and self-compassion levels has been found previously, suggesting that the frequency and length of a yoga intervention plays a crucial role in achieving optimal changes in mindfulness and self-compassion scores (23).

Mind-body interventions such as yoga are increasingly used for stress reduction, often investigated with the perceived stress scale (22, 24, 49). In a systematic review addressing the effects of yoga on stress in healthy individuals, yoga was beneficial on reducing stress levels in the majority of the included studies (50). However, here we did not observe a decrease in perceived stress. Some participants mentioned at the end of the study that introducing the intervention in their daily life schedule was even stressful at times. Other studies have found even increased stress levels at the end of a yoga intervention that could be related to a too overwhelming class schedule on top of daily activities and/or that the expectations were not in line with reality (49). As previous research has addressed a beneficial effect of yoga in mood disorder (depression and anxiety), we also explored the effects of yoga on affect in healthy subjects. In this study, we found increased scores on cognitive and overall functioning with the LAPS in the yoga group compared to the controls after the intervention. Although no differences were found on positive affect, increases in cognitive functioning may precede effects on positive affect, as a link between cognitive functioning and positive affect has been described previously (27).

Regarding the three neuroimaging markers (i.e., glucose metabolism, synaptic density, and GM volume), we did not observe significant differences in any of these markers after the yoga intervention compared to baseline. In our previous cross-sectional study, a strong and highly significant decrease in the medial temporal cortex, striatum, and brainstem was observed in experienced yoga subjects, with an average of 4.8 years of yoga experience and at least four practices per week, totaling at least 150 sessions per year (10). This is markedly different from the frequency and duration of the current study where yoga-naive participants practiced yoga only twice a week for 12 weeks with on average 21 sessions, which may have been too short or too infrequent to instigate measurable metabolic effects. The intervention duration for the current study was chosen as a practically achievable time scale, so new designs will have to take longer durations and/or more frequent sessions into consideration.

After the indoor cycling intervention, significant clusters of increased resting glucose metabolism were found in cognitive and motor brain areas: parahippocampal gyrus, fusiform gyrus, and upper cerebellum. The parahippocampal gyrus is associated with many cognitive processes, including visuospatial processing and episodic memory, but also emotion processing (51). Extensive involvement of the parahippocampal gyrus has been found in the majority of animal and human studies investigating neuronal activity in relation to physical activity (52), and was associated with positive effects on memory (53, 54). This is supported by evidence of increased levels of serum brain derived neurotrophic factor (BDNF) after exercise interventions (55–57). Brain derived neurotrophic factor is an important molecule for synaptic plasticity and known to play a crucial role in learning and memory (58). Increased levels of BDNF have been repeatedly reported in the hippocampal regions of rodents after physical activity (59, 60). Similarly, elderly healthy subjects that were more physically active showed higher glucose metabolism in the parahippocampus and fusiform gyrus (61). The link between physical activity and increased glucose metabolism in the fusiform gyrus remains speculative as the fusiform gyrus is mainly known for its involvement in functionally specialized computations of higher-order visual features such as object recognition and face perception (62, 63). In line with the observed increased glucose metabolism in the upper cerebellum, Talukdar et al. found that general aerobic fitness was significantly associated with increased brain activity in the upper cerebellum in addition to sensory, motor, and memory processing regions (64). The connections of the upper cerebellum with the primary motor cortex and a somatotopic organization of both lower and upper limbs, play a pivotal role in motor functioning, which is heavily interrogated during indoor cycling or aerobic activity (more than in slow, postural changes in yoga) and could therefore explain this association.

It can be hypothesized that alterations in glucose metabolism, a main determinant of synaptic activity, may after time lead to strengthening of synapses (65). Therefore, only after long enough metabolic activation, more synaptic connections (hence synaptic density) and ultimately microstructural increases in GM volume can be expected. Concerning effects of yoga on macroscopic structure, to the best of our knowledge only two studies have been published in a longitudinal interventional setting. Also here, despite daily practicing yoga (mindfulness based stress reduction including yoga postures and yogic meditation) over a period of 8–12 weeks, no increased GM volume was found (66, 67), (which is) in line with our data. In addition, interventional longitudinal imaging studies investigating physical activity in healthy subjects are limited as well. Erickson et al. found that in healthy elderly individuals hippocampal volumes increased after regular aerobic exercise at moderate-intensity for 1 year (68). Another interventional study in healthy elderly individuals before and after 6 months of aerobic training found increased volumes in the anterior cingulate cortex, supplementary motor association cortex, inferior frontal gyrus, and left superior temporal gyrus (69). Thus, only in studies conducted over a longer time span and higher cumulated activity levels compared to our study, significant effects were found.

Additionally, no changes in synaptic density were found using [^11^C]UCB-J PET imaging in the subcohort. Even a more liberal threshold up to 0.01 did not result in significant clusters. As the field of *in vivo* synaptic density imaging is still very young (12), no other studies investigating the effects of any behavioral/physical interventions on synaptic density in humans have been published so far. Therefore, it is difficult to speculate on the intensity of a behavioral intervention that would be needed for detection of a macroscopic difference combined to the test sensitivity (T-RT values about 5%) (70). In rodents, aerobic exercise (treadmill running), 5 days a week for 4 or 12 weeks resulted in increased hippocampal synaptic density (60.6–75.1% higher number of synapses per cubic micron of tissue in treadmill training vs. sedentary), using post-mortem immunofluorescent staining and electron microscopy (71, 72).

A few study limitations should be addressed. First, the group size in this academic interventional trial was relatively small and the duration and frequency of the intervention was limited, because the feasibility of the study for the subjects was considered. However, previous studies investigating effects of yoga on the brain used similar study designs in terms of study duration and frequency (4, 66, 67, 73–75). The study length and frequency were considered as an optimal balance between study feasibility, duration, cost, and potential drop-out rate. Secondly, as no significant differences in GM volume between both groups, nor between the baseline vs. the post- intervention condition, were found, we did not apply partial volume correction on the PET data. Thirdly, the participants were randomly assigned to either the yoga intervention or the indoor cycling intervention. As subjects may have showed preference toward one specific intervention, and preference of the participant may influence the outcome (76), a chance of underperforming in the control group may have occurred. However, based on the number of sessions followed and the detailed, consistent information in self-reporting logs of the participants in both groups, we expect no systematic bias from this aspect. Finally, the yoga group could attend classes of four different yoga styles, each having a different emphasis. Although the relative composition of the yoga classes (postures/breathing/meditation) remained stable, there is still debate about which component of yoga causes the most substantial behavioral or physical benefits (7). Thus, a different ratio between the components could be necessary to detect stronger neuronal effects. Furthermore, these types of comprehensive changes may require longer time before macroscopic effects become evident.

In conclusion, we found that a yoga intervention of 12 weeks increases interoceptive awareness. However, we were not yet able to observe metabolic, synaptic density or volumetric correlates of this behavioral finding after this short intervention. Therefore, in line with previous results, longer interventions and/or higher frequency of yoga practice may be needed to objectivate cerebral metabolic and/or structural brain effects. Furthermore, indoor 12 weeks of cycling did significantly increase regional glucose metabolism in brain regions important for motor functioning and cognition, but did not result into interoceptive or measurable cognitive improvements.

## Data Availability Statement

The raw data supporting the conclusions of this article will be made available by the authors, without undue reservation.

## Ethics Statement

The studies involving human participants were reviewed and approved by local University Ethics Committee of Leuven. The patients/participants provided their written informed consent to participate in this study.

## Author Contributions

JvA, KVL, and KD contributed to conception and design of the study. JvA, LJ, and KVL collected the data. JvA, LJ, JC, SS, MK, and KVL performed the (statistical) analysis. JvA wrote the first draft of the manuscript. JvA, LJ, JC, KD, SS, and KVL wrote sections of the manuscript. All authors contributed to manuscript revision, read, and approved the submitted version.

## Funding

KVL is senior clinical research fellow for the Research Foundation-Flanders (FWO), JC is post-doctoral fellow for FWO.

## Conflict of Interest

The authors declare that the research was conducted in the absence of any commercial or financial relationships that could be construed as a potential conflict of interest.

## Publisher's Note

All claims expressed in this article are solely those of the authors and do not necessarily represent those of their affiliated organizations, or those of the publisher, the editors and the reviewers. Any product that may be evaluated in this article, or claim that may be made by its manufacturer, is not guaranteed or endorsed by the publisher.
