# Supplementary material for: Twelve-Week Yoga vs. Aerobic Cycling Initiation in Sedentary Healthy Subjects: A Behavioral and Multiparametric Interventional PET/MR Study
Source: Front Psychiatry. 2021 Oct 18;12:739356. doi: 10.3389/fpsyt.2021.739356 (PMC8558251; doi:10.3389/fpsyt.2021.739356)
Supplement: Supplementary file 1 [file Data_Sheet_1.docx]

# Supplementary Tables

**Supplementary TABLE 1.** Statistical parametric mapping analysis for increased relative glucose metabolism in the cycling group post intervention, compared to pre. Contrast: pre < post in cycling.

|  |  |  | Voxel |  | Peak voxel MNI | | | |  | Cluster peak intensity |  |  |
| --- | --- | --- | --- | --- | --- | --- | --- | --- | --- | --- | --- | --- |
| Cluster level | |  | level |  | coordinates | | | |  | Difference |  | Cluster location |
| *P*_FWE_ | K_EXT_ |  | *t* |  | x | y | | z |  | (%) |  |  |
| 0.001 | 3.2 |  | 7.86 |  | 24 | -34 | -33 | |  | 4.3 |  | Cerebellum 4 5 |
|  |  |  | 5.82 |  | 32 | -32 | -26 | |  |  |  | Fusiform gyrus |
|  |  |  | 5.21 |  | 22 | -24 | -24 | |  |  |  | Cerebellum 4 5 |
|  |  |  | 4.81 |  | 34 | -15 | -30 | |  |  |  | Fusiform gyrus |
|  |  |  | 4.70 |  | 40 | -14 | -36 | |  |  |  | Fusiform gyrus |
|  |  |  | 4.55 |  | 30 | -20 | -28 | |  |  |  | Parahippocampus |
|  |  |  | 4.52 |  | 24 | -10 | -32 | |  |  |  | Parahippocampus |
|  |  |  | 4.18 |  | 24 | -12 | -27 | |  |  |  | Hippocampus |
| 0.002 | 2.9 |  | 6.08 |  | -30 | -33 | -30 | |  | 5.7 |  | Cerebellum 4 5 |
|  |  |  | 5.75 |  | -38 | -32 | -22 | |  |  |  | Fusiform gyrus |
|  |  |  | 5.59 |  | -30 | -26 | -24 | |  |  |  | Parahippocampus |
|  |  |  | 4.39 |  | -22 | -28 | -20 | |  |  |  | Parahippocampus |
|  |  |  | 4.30 |  | -28 | -18 | -27 | |  |  |  | Parahippocampus |

K_EXT_, cluster size extent (cm^3^); MNI, Montreal Neurological Institute; *P*_FWE_, familywise error corrected *P*-value.

# Supplemental Figures


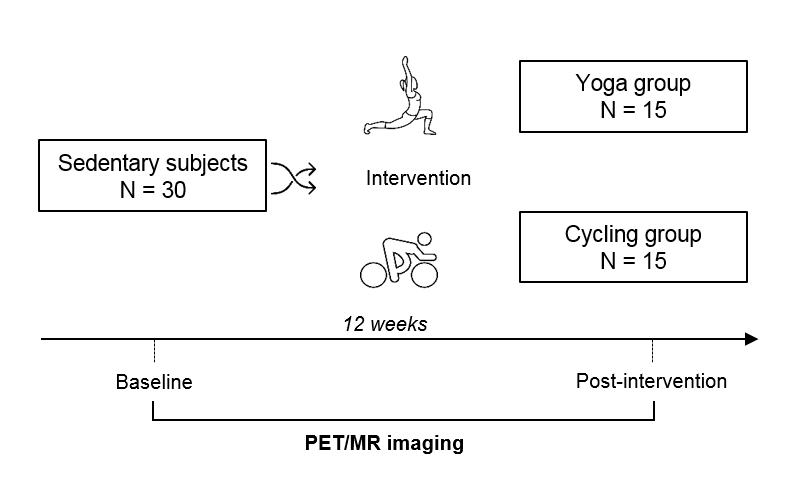


**Supplementary Figure 1** study design. Sedentary subjects were randomly assigned to either the yoga (yoga group) or the indoor cycling (cycling group) intervention after their baseline scan. PET/MR images were acquired at baseline and post-intervention. Psychometric scales were sampled at the same time points.
